# Poly(acrylic acid)/Poly(vinyl alcohol) Microarray Patches for Continuous Transdermal Delivery of Levodopa and Carbidopa: In Vitro and In Vivo Studies

**DOI:** 10.3390/pharmaceutics16050676

**Published:** 2024-05-17

**Authors:** Yaocun Li, Lalitkumar K. Vora, Jiawen Wang, Akmal Hidayat Bin Sabri, Andrew Graham, Helen O. McCarthy, Ryan F. Donnelly

**Affiliations:** School of Pharmacy, Queen’s University Belfast, 97 Lisburn Road, Belfast BT9 7BL, UK; yli47@qub.ac.uk (Y.L.); l.vora@qub.ac.uk (L.K.V.); jwang28@qub.ac.uk (J.W.); akmal.binsabri1@nottingham.ac.uk (A.H.B.S.); agraham69@qub.ac.uk (A.G.); h.mccarthy@qub.ac.uk (H.O.M.)

**Keywords:** dissolving microarray patch, levodopa, carbidopa, transdermal drug delivery

## Abstract

Levodopa (LD) has been the most efficacious medication and the gold standard therapy for Parkinson’s disease (PD) for decades. However, its long-term administration is usually associated with motor complications, which are believed to be the result of the fluctuating pharmacokinetics of LD following oral administration. Duodopa^®^ is the current option to offer a continuous delivery of LD and its decarboxylase inhibitor carbidopa (CD); however, its administration involves invasive surgical procedures, which could potentially lead to lifelong complications, such as infection. Recently, dissolving microarray patches (MAPs) have come to the fore as an alternative that can bypass the oral administration route in a minimally invasive way. This work explored the potential of using dissolving MAPs to deliver LD and CD across the skin. An acidic polymer poly(acrylic acid) (PAA) was used in the MAP fabrication to prevent the potential oxidation of LD at neutral pH. The drug contents of LD and CD in the formulated dissolving MAPs were 1.82 ± 0.24 and 0.47 ± 0.04 mg/patch, respectively. The in vivo pharmacokinetic study using female Sprague–Dawley^®^ rats (Envigo RMS Holding Corp, Bicester, UK) demonstrated a simultaneous delivery of LD and CD and comparable AUC values between the dissolving MAPs and the oral LD/CD suspension. The relative bioavailability for the dissolving MAPs was calculated to be approximately 37.22%. Accordingly, this work highlights the use of dissolving MAPs as a minimally invasive approach which could potentially bypass the gastrointestinal pathway and deliver both drugs continuously without surgery.

## 1. Introduction

Parkinson’s disease (PD) can occur at various stages of life, with a high risk among elderly people, and its prevalence has led to a global healthcare issue associated with progressive neurodegenerative disorders, with approximately 10 million people being affected globally [1]. The neurobiological basis of PD is poorly understood; however, it is a widely held view that the depletion of dopaminergic neurons in the substantia nigra pars compacta and the distribution of α-synuclein-immunoreactive proteins known as Lewy body inclusions can give rise to PD-associated movement disorders [1,2,3]. Therefore, the restoration of nigrostriatal dopamine-triggered signalling pathways has a pivotal role in suppressing PD-associated symptoms.

Over the last several decades, a naturally occurring amino acid, levodopa (LD), formulated together with a peripheral decarboxylase inhibitor, carbidopa (CD), has been considered the gold standard therapy for PD [3,4]. The exact underlying mechanism of its action is not clearly understood, but the conversion from LD to dopamine within the striatum can replenish dopaminergic deficiency associated with the PD patients [5]. While effective, one major issue in long-term PD treatment is LD-induced motor complications, such as rapid fluctuations between good and poor symptom management [4,6,7]. The aetiology of such drug-induced side effects was believed to be the fluctuating pharmacokinetic profile, and thus the intermittent delivery, of LD [3,4]. Once orally administered, LD undergoes extensive first-pass metabolism and rapid distribution into the skeletal muscles. The competence of transport carriers from other amino acids, which varies upon daily food uptake, and the delayed gastric emptying time in PD patients results in a frequent treatment ‘off state’ and, more importantly, an insufficient amount of LD delivered to the brain [8,9]. It is also challenging for a PD patient to adhere to a treatment regimen that requires administration at several time points within a single day [10]. To date, there have been a range of clinical investigations aiming to address the fluctuating pharmacokinetic profile as well as the ’off’ periods between consecutive doses, including controlled-release oral formulations, inhaled formulations, intestinal formulations, and subcutaneous infusion systems [11]. The clinical benefits, in terms of reducing ‘off’ periods, of using controlled-release tablets such as Sinemet^®^ are not considered marked [12]. Inhalation-based LD formulations can facilitate drug absorption via the pulmonary system and therefore bypass the gastrointestinal tract [13]. There are no currently marketed inhalable LD products in the United Kingdom. Such an approach holds potential to improve the pharmacokinetic profile of LD, whereas some practical aspects, including the dose control and powder-induced coughing, should be addressed to progress the formulations to the market. An LD/CD intestinal gel (Duodopa^®^) has been developed to bypass gastric emptying in the stomach and facilitate continuous LD/CD delivery, which can provide a more stable plasma concentration than oral tablets [14]. Such a formulation presents an alternative treatment option when motor fluctuations occur with oral treatment. However, it requires an invasive procedure, where LD/CD is delivered via a jejunal percutaneous endoscopic gastrostomy (PEG-J) tube connected to a pump, and PEG-J tube-related complications (e.g., peristomal infection) have been quite commonly reported [15]. Therefore, seeking a non-invasive or minimally invasive alternative formulation has been a pressing issue in the field of PD treatment [4].

Microarray patches (MAPs), sometimes known as microneedle patches, are minimally invasive transdermal drug delivery devices that have garnered a great deal of attention in the last decade [16,17,18]. The avoidance of first-pass metabolism in this transdermal approach presents itself as a promising approach for therapeutics with significant first-pass metabolism [19]. MAPs are able to pierce through the outermost layer of the skin, the stratum corneum (SC), which is the main physiological barrier of the skin, and subsequently reach the dermal microcirculation [20]. Additionally, compared to conventional parenteral formulations, MAPs could potentially improve treatment adherence by avoiding pain perception, given the microscale needle size, especially for patients with needle phobia issues [16,21]. Among the five available MAP drug delivery systems, dissolving MAPs hold great potential for the continuous delivery of both hydrophilic and hydrophobic compounds by forming an intradermal drug depot, further releasing therapeutics into the rich dermal microcirculation [21]. Dissolving MAPs are generally fabricated using water-soluble, biodegradable, or biocompatible polymeric materials. Following insertion into the skin, the polymeric matrix of the dissolving MAP rapidly dissolves and leaves the incorporated therapeutic agents in the skin dermal layer [22]. The self-disabling nature of such an approach also greatly reduces any risk associated with needle reuse cross-contamination, as well as sharp waste [23]. Furthermore, the drug release could be controlled by using different water-soluble polymers in the MAP matrix. Accordingly, dissolving MAPs have been widely investigated for the transdermal delivery of a broad range of low-molecular weight therapeutics, vaccines, and DNA [24].

Previous work has demonstrated a simultaneous delivery of LD and CD using hydrogel-forming MAPs, whereas no drug could be detected for the first 6 h and no continuous release was observed [25]. It is ideal to have a rapid onset of action for a PD treatment option. In this work, a dissolving MAP formulation loaded with LD and CD, which could potentially dissolve rapidly upon insertion into the skin and facilitate a continuous release, was developed and investigated in terms of the pharmacokinetics in comparison to those of an oral suspension. The dissolving MAPs were mechanically characterised and then assessed ex vivo for their facilitation of permeation across dermatomed porcine skin obtained from stillborn piglets. The in vivo pharmacokinetic profiles of the formulated dissolving MAPs were evaluated using healthy Sprague–Dawley^®^ rats. This is the first time that the use of dissolving MAPs has been justified for the transdermal delivery of both LD and CD simultaneously with a continuous release behavior. Furthermore, this approach also highlights the prospect of bypassing the unpredictable gastric mobility of PD patients in a minimally invasive way.

## 2. Materials and Methods

### 2.1. Materials

LD (molecular weight = 197.19 Da) and CD (molecular weight = 226.23 Da) were supplied by TEVA Pharmaceuticals (Petah Tikva, Israel). Poly(vinyl alcohol) (PVA) (MW = 9–10 kDa, 80% hydrolysed), poly(acrylic acid) (PAA) (MW = 250 kDa) 35% *w*/*w* solution, poly(vinyl pyrrolidone) (PVP) (MW = 360 kDa), and PVP (MW = 58 kDa) were purchased from Sigma-Aldrich (Dorset, UK). Glycerol AnalaR NORMAPUR (purity 99.5%) was purchased from VWR International UK (Leicestershire, UK). Xiameter^®^ RTV-4250-S mould-making silicone base and RTV-4250-S curing agent were purchased from Notcutt (Surrey, UK). All the other reagents used are of analytical grade.

### 2.2. Preparation of Silicone Ring for MAP Casting

**Figure 1:**
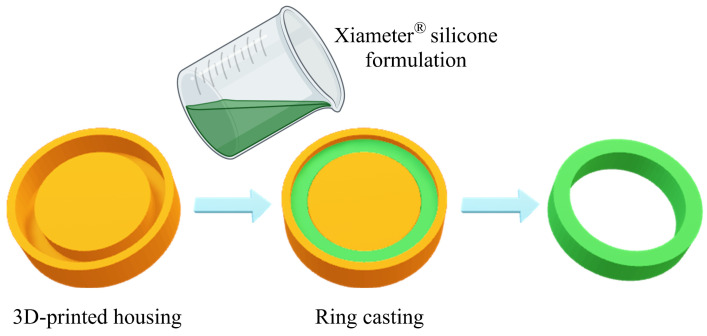
A schematic illustration of ring insert preparation for ‘two-layer’ dissolving MAP casting.

Silicone rings that can be attached to the surface of MAP moulds to prevent formulation spilling during centrifugation were fabricated as previously described [26]. A master template for the silicone ring was first designed using Tinkercad (Autodesk, Helsinki, Finland) and then 3D-printed using acrylonitrile butadiene styrene (ABS) filaments via an Ultimaker^®^ 3 3D printer (Ultimaker B.V., Geldermalsen, The Netherlands). The Xiameter^®^ silicone base and its corresponding curing agent were mixed at a ratio of 10:1, then blended until homogeneous using a Speedmixer^TM^ (Synergy Devices Ltd., London, UK) at 3000 rpm for 15 s. The resulting formulation was then poured into the 3D-printed master template and left at ambient temperature overnight to ensure complete curing, as shown in Figure 1.

### 2.3. Effect of Polymer Addition on LD/CD Chemical Stability

The impact of polymer solution addition on the chemical stability of LD and CD was investigated by analysing the drug content of the LD/CD formulation that would be used for MAP casting. For this purpose, instead of casting the formulation into dissolving MAP moulds, a polymeric film was prepared for quantification via high performance liquid chromatography coupled with an ultraviolet detector (HPLC–UV), as described in Section 2.6.1. Aqueous blends of the MAP ‘drug-containing layer’ formulation (40 g) (48% *w*/*w* LD/CD, 1.6% *w*/*w* PAA 250 kDa, 30.4% *w*/*w* PVA 9–10 kDa, 20% *w*/*w* deionised water) were centrifuged at 4000 rpm for 10 min to exclude air bubbles and then homogeneously spread onto the surface of a silicone release liner sitting on a 10 × 10 cm square film mould. The film was allowed to dry at ambient room temperature for 48 h and removed from the mould after drying. The film was trimmed into 1 cm^2^ squares and transferred into a vacuum desiccator to avoid introducing moisture to the system. At predetermined time points (0, 3, 6 weeks), the film was dissolved and the drug content was analysed. The percentage recovery was calculated using Equation (1).

(1) $$Recovery\;\left(\%\right)=\frac{\frac{drug\;content\;in\;the\;film}{total\;film\;mass}}{initial\;drug\;content\;per\;unit\;film\;area}*100\%$$

### 2.4. Fabrication of Dissolving MAPs Loaded with LD and CD

**Figure 2:**
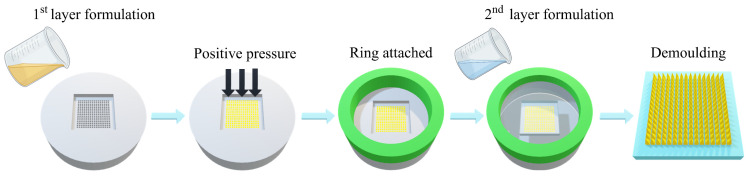
A schematic illustration of ‘two-layer’ dissolving MAP casting.

For the formulations involved in LD/CD dissolving MAP fabrication, LD and CD were mixed in a mass ratio of 4:1, as indicated in the current Rytary^®^ marketed tablet [4]. An aqueous blend investigated in Section 2.3 was mixed with the aid of a Speedmixer^TM^ at 3000 rpm for 5 min. The resulting formulation was then subjected to MAP casting as shown in Figure 2. The pH values of the polymer blends were measured using a digital pH meter for stability considerations. The baseplate formulation was fabricated by attaching the silicone ring fabricated in Section 2.2 and adding aqueous blends of 25% *w*/*w* PVP 360 kDa and 1.5% *w*/*w* glycerol. An extra 1% *w*/*w* citric acid was added to maintain an acidic pH for stability considerations. The MAP moulds were then centrifuged at 4000 rpm for 5 min and the rings were removed afterwards. After 24 h of drying at ambient temperature, MAPs were trimmed into 1 cm^2^ squares and stored in a vacuum desiccator for further characterisation.

### 2.5. Characterisation of Fabricated Dissolving MAPs

#### 2.5.1. Visual Characterisation of Fabricated Dissolving MAPs

The morphologies of the fabricated dissolving MAPs were examined using a Leica EZ4D digital microscope (Leica Microsystems, Milton Keynes, UK). A Keyence digital microscope (Keyence, Osaka, Japan) was used for high-resolution imaging in glare-removal mode.

#### 2.5.2. Physical Characterisation of Formulated Dissolving MAPs

Physical characterisations of dissolving MAPs included mechanical strength, investigated as the height reduction post-compression against an aluminum platform with 32 N pressure for 30 s, and insertion abilities, investigated as the holes created in an artificial Parafilm M^®^ membrane described in a previously proposed. The force (32 N) was the maximum average human force for MAP insertion determined by previous work, and the time (30 s) was suggested in a previous human insertion study [27,28,29]. A TA.XT texture analyser (Stable Micro Systems, Surrey, UK) attached to a cylindrical probe (cuboidal base, 10 × 10 mm) was programmed in compression mode with a speed of 1.19 mm/s (moving downwards) until the force was reached. The needle height after compression and the holes created in each Parafilm^®^ layer were measured via the Leica EZ4D digital microscope for mechanical strength and insertion ability characterisations, respectively. Equations (2) and (3) were used for data calculation and presentation.

(2) $$Percentage\;height\;reduction\;\left(\%\right)=\left(\frac{Hi-Ha}{Hi}\right)*100$$

(3) $$Percentage\;holes\;created\;\;\left(\%\right)=\left(\frac{Number\;of\;holes\;created}{Total\;needle\;number}\right)*100$$

#### 2.5.3. Dissolution Studies of Formulated Dissolving MAPs

The dissolution of individual needles after insertion was investigated using full-thickness neonatal porcine skin. Porcine skin shares similar physiology and anatomy to human skin and has been reported as a suitable ex vivo skin model [30,31]. Full-thickness skin was excised using a scalpel blade and carefully shaved using a disposable razor. After equilibration in phosphate-buffered saline (PBS) at pH 7.4 for 20 min, MAPs were inserted via the TA.XT texture analyser as described in Section 2.5.2. The inserted MAPs and skin were then placed in a weighing boat containing PBS-soaked absorbent paper and transferred to a 37 °C oven. At 15 min, 30 min, 45 min, and 60 min, the MAPs were removed from the skin and the remaining needle length was measured with the Leica EZ4D digital microscope.

#### 2.5.4. Determination of Dissolving MAPs’ Drug Content

Each dissolving MAP had a 0.5 cm^2^ drug-containing area and was dissolved in 5 mL of 0.1 N hydrochloric acid (HCl) in a 7 mL glass vial in this study. After complete dissolution, the glass vial was vortexed for 10 s, and 100 μL of the vortexed sample was immediately withdrawn and diluted by a factor of 10. The diluted samples were then centrifuged at 14,800 rpm for 15 min and subjected to HPLC–UV analysis.

#### 2.5.5. Thermogravimetric Analysis

Thermogravimetric analysis (TGA) was applied to quantify the water content of the fabricated dissolving MAPs and investigate the effectiveness of the drying process. Needle tips of MAPs were scraped off carefully with a scalpel blade and collected in a 7 mL glass vial. An appropriate number of needle samples was added into an aluminum pan to fill in the cavity. A heating rate of 10 °C/min was applied to heat the samples from 20 °C to 300 °C. Nitrogen balance purge and sample purge gases were maintained at 40 mL/min and 60 mL/min, respectively. The initial weight was recorded and analysed using TA Instruments Universal Analysis 200 software (version 4.4a).

#### 2.5.6. Ex Vivo Skin Permeation Studies Using Dermatomed Neonatal Porcine Skin

Ex vivo permeation profiles of LD/CD dissolving MAPs were investigated using a modified PermeGear Franz cell apparatus [26,32]. Briefly, dermatomed neonatal porcine skin (~350 μm thickness) was carefully shaved and placed between the donor and receiver compartment. Dissolving MAPs were then inserted into the dermatomed skin fixed onto the Franz cell donor using cyanoacrylate glue with manual force for 30 s, and a 15 g cylindrical aluminium weight was placed on top of the MAP to maintain the in situ position. The diameter of both the donor and receiver compartment orifices was 15 mm. PBS with 0.1 N HCl, the release media for ex vivo permeation studies selected based on previous work, was added into the receiver compartment [25]. At predetermined time points, 200 µL aliquots were collected, analysed through the HPLC–UV method, and replaced with fresh release media over a time period of 24 h. The apparatus was kept at 37 ± 1 °C to mimic in vivo conditions. The experiments were carried out in triplicate.

#### 2.5.7. In Vivo Pharmacokinetic Studies Using Healthy Sprague–Dawley^®^ Rats

Healthy female Sprague–Dawley^®^ rats (12 weeks aged, 218 ± 13 g, *n* = 12) were purchased from Envigo (Envigo RMS Holding Corp, Bicester, UK) and used for in vivo pharmacokinetic evaluation of fabricated dissolving MAPs. Study protocols were designed under Project License 2903 and approved by the committee of the Biological Service Unit (BSU) at Queen’s University Belfast. PILs 2175, 1892, and 2127 were involved in this study. The experimental procedure was carefully designed and performed to implement the principles of replacement, reduction, and refinement (3Rs) throughout the study.

**Figure 3:**
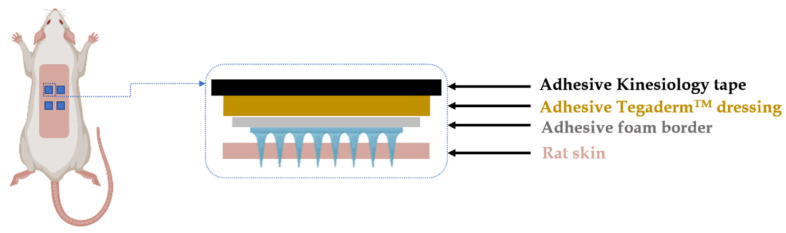
A schematic illustration of MAP application to rats in in vivo pharmacokinetic studies.

The hair on the rat skin was carefully shaved using an electric razor and hair removal cream (5 min) 24 h before the MAP application to minimize the influence of relatively high hair density on MAP insertion. For the dissolving MAPs cohort, each rat received four dissolving MAPs with a specific ‘wrapping’ to prevent rats from displacing and consuming the applied MAPs, as illustrated in Figure 3. Briefly, an adhesive foam border layer, an adhesive Tegaderm^TM^ (3M, Bracknell, UK) dressing layer, and adhesive kinesiology tape were placed on top of the inserted MAPs to maintain their position. The dose given to the rats by the dissolving MAPs was determined based on ex vivo bioavailability and matched with the control cohort. Table 1 lists the experimental design of this in vivo pharmacokinetic study.

#### 2.5.8. Interpretation of MAP Relative Bioavailability

Relative bioavailability refers to the systemic availability of a given formulation compared to that of the control/reference formulation, in this case, the bioavailability of the MAP versus the oral bioavailability of LD. Therefore, the bioavailability of the MAP was calculated using Equation (4), where F_oral_ is the bioavailability of the formulation utilised in the control cohort [33]. AUC values were calculated based on the pharmacokinetic profiles obtained from the dissolving MAPs and the oral gavage cohorts.

(4) $$F\left(MAP\right)/F(Oral)=\left(\frac{AUC\;\left(MAP\right)*Dose\;\left(oral\right)}{AUC\;(oral)*Dose\;\left(MAP\right)}\right)$$

### 2.6. Pharmaceutical Analysis

#### 2.6.1. In Vitro and Ex Vivo Sample Analysis via HPLC–UV

In vitro and ex vivo samples of LD and CD were analysed via an Agilent 1200^®^ (Agilent Technologies Ltd., Stockport, UK) HPLC–UV system. An APEX Inertsil^®^ (GL Sciences Inc., Tokyo, Japan) ODS-3 column (5 μm particle size, 250 × 4.6 mm) was selected as the stationary phase for separation at ambient temperature, and the mobile phase was composed of 20:80% *v/v* methanol and potassium dihydrogen monophosphate buffer (25 mM, pH adjusted to 3 using phosphoric acid) at a flow rate of 0.8 mL/min. Vacuum filtration was applied for mobile phase degassing. UV detection was set at 280 nm, and the injection volume was 50 μL. The total run time was 8 min, and the retention times for LD and CD were 3.9 min and 5.4 min, respectively. Agilent Chemstation^®^ version 3.5.0 was used for chromatogram analysis.

#### 2.6.2. In Vivo Sample Analysis via HPLC–Fluorescence

A liquid precipitation extraction method was utilised to process LD/CD plasma samples. Prior to extraction, 5 μL of sodium metabisulfite (80 mg/mL), an antioxidant, was added to each of the Eppendorf^®^ tubes. Perchloric acid (1 M, 50 μL) was then added into the mixture to precipitate the plasma interferences and vortexed for 30 min at 1500 rpm. A homogenised mixture of sodium metabisulfite, LD, CD, perchloric acid, and plasma was then centrifuged at 15,300 rpm for 20 min at 4 °C using a Sigma^®^ 2–16 K refrigerated centrifuge (Sigma Centrifuges, Osterode am Harz, Germany). The supernatant (80 μL) was sampled from the Eppendorf^®^ tube and transferred into an Agilent^®^ 250 μL vial insert, which was placed into a HPLC vial and then injected into the HPLC for analysis.

In vivo sample analysis of LD/CD was achieved via an Agilent 1200^®^ HPLC coupled with a fluorescence detector. The same column (the APEX Inertsil^®^ ODS-3 column) used in the in vitro and ex vivo sample analyses was used for in vivo sample analyses. Chromatographic separation was performed at ambient temperature, with a mobile phase composition of 10:90% *v/v* methanol and potassium dihydrogen monophosphate buffer (25 mM, pH adjusted to 3 using phosphoric acid) at a flow rate of 1.2 mL/min. The mobile phase was degassed by vacuum filtration prior to use. Fluorescence detection was conducted at an excitation wavelength of 280 nm and an emission wavelength of 310 nm. The injection volume was 50 μL. The total run time was 15 min, and the retention times for LD and CD were 3.8 min and 6.8 min, respectively. The obtained chromatograms were analysed with the aid of Agilent Chemstation^®^ version B.04.02.

### 2.7. Statistical Analysis

Data mean and standard deviation (S.D.) values were calculated using Microsoft^®^ Excel^®^ 2022 (Microsoft Corporation, Redmond, WA, USA). Statistical analysis was performed using GraphPad Prism^®^ (version 9.0.0) (GraphPad Software, San Diego, CA, USA). For two unpaired datasets, a Mann–Whitney U test was applied, and a Kruskal–Wallis test was performed for three and more data groups. Unless otherwise stated, a significance level of *p* < 0.05 was applied to all statistical analyses.

## 3. Results and Discussion

### 3.1. Stability Investigation of the LD/CD MAP ‘Drug-Containing’ Layer

Given that LD can be easily oxidised, it is crucial to prevent degradation and drug–polymer interaction during manufacture where possible [34]. A strong relationship between pH and LD oxidation has been reported in the literature. The conversion from colourless hydroquinone to coloured quinones is pH dependent, and a pH range between 2 and 4 demonstrated a stable LD concentration over at least 50 h, indicating the necessity of controlling the MAP formulation pH [35]. An acidic biodegradable polymer, PAA (Mw 250 kDa) has been widely utilised for dissolving MAP manufacturing and demonstrates rapid dissolution upon insertion in spite of its relatively high molecular weight [36,37]. Therefore, PAA was used for the ‘drug-containing’ layer casting, and 1% *w*/*w* citric acid was added in the baseplate formulation to main an acidic pH during MAP fabrication. The formulation parameters are summarised in Table 2.

**Figure 4:**
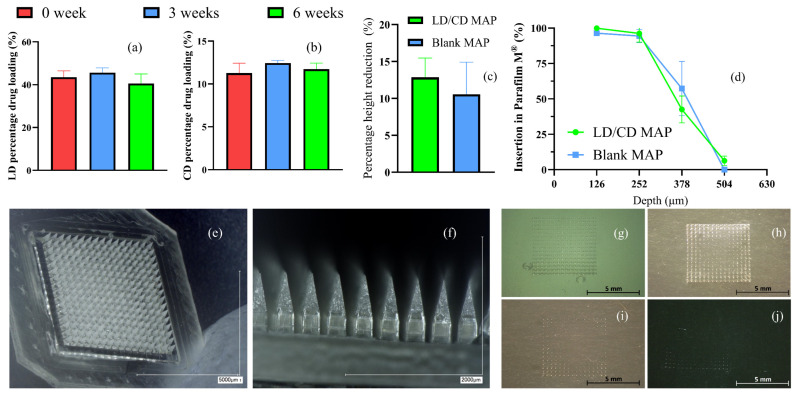
(**a**) LD content and (**b**) CD content in the LD/CD MAP ‘drug-containing layer’ formulation (means + S.D., *n* = 3). (**c**) Percentage height reduction in MAP needles upon 32 N/cm^2^ pressure for 30 s (means + S.D., *n* = 5). (**d**) Insertion profiles of dissolving MAPs (means ± S.D., *n* = 3). (**e**) Representative digital microscopic images of the LD/CD dissolving MAPs. (**f**) Drug distribution of the LD/CD dissolving MAPs. Representative microscopic images of the (**g**) first layer, (**h**) second layer, (**i**) third layer, and (**j**) fourth layer of Parafilm M^®^ in the insertion studies.

The stability of the LD/CD formulation during the MAP fabrication process was evaluated by investigating the drug content of a polymer film that was prepared using the formulation utilised for LD/CD ‘drug-containing layer’ casting. Aluminum sealing was applied to exclude the influence of light upon oxidation. As depicted in Figure 4a, comparing the LD drug content on day 0 (43.48 ± 2.73%) with that at 3 weeks (45.61 ± 2.08%) and 6 weeks (40.53 ± 4.07%), there was no significant drug content difference over a time period of 6 weeks (*p* = 0.0590), which suggested an optimal formulation pH and a minimal impact of polymer addition on LD stability. The CD content also remained unchanged over a time period of 6 weeks (*p* = 0.0720). The result demonstrated that the use of PAA provided an acidic environment which could prevent the oxidation of LD and CD, which was in accordance with the previously reported LD/CD optimal pH range (2–4) to maintain good chemical stability [35].

### 3.2. Visual Characterisations of Formulated LD/CD Dissolving MAPs

A Keyence digital microscope was used to visualise and examine the morphologies of the formulated LD/CD dissolving MAPs. Since dissolving MAPs are designed to facilitate drug delivery by penetrating the SC and depositing drug in the skin viable layers, the products should not only possess sufficient mechanical properties for insertion but also a high capacity for drug incorporation. The geometry selected in this work, a cuboidal base and pyramidal needle top, were described in a previous work by Cordeiro et al. in which the design demonstrated an approximately three times higher drug loading capacity in comparison to conventional geometries (e.g., full conical shape) without compromising the mechanical properties of the MAPs [38]. Sharp needles such as pyramidal or conical shapes have been found with lower insertion force required to reach the dermal layer [39]. Additionally, the cuboidal base at the bottom affords greater available space for drug loading, which is particularly important at the preliminary stage of formulation screening. This design has consequently been widely utilised in research concerning dissolving MAP-mediated transdermal delivery of both hydrophilic and hydrophobic compounds [40,41,42]. In terms of the drug distribution, the localisation of drug formulations at the needle tip instead of the supporting baseplate further reduces unnecessary drug wastage, as the part of the needles inserted beneath the SC is most likely available for drug delivery [41,43,44]. Individual needles were found to be intact, sharp, and uniformly distributed across the patch, as illustrated in Figure 4c,d. A ‘two-layered’ structure was also revealed, where the grey crystal-like upper portion of the needle indicated the ‘drug-containing’ layer and the transparent baseplate indicated the ‘blank’ supporting layer.

### 3.3. Physical Characterisations of the Formulated LD/CD Dissolving MAPs

The physical properties of the formulated dissolving MAPs were characterised in terms of their height reduction against pressure and insertion depth in a previously developed Parafilm^®^ M insertion model [28]. The needle length after compression and holes created in each Parafilm M^®^ layer were visually examined with a Leica EZ4D digital microscope. Generally, needles with a height reduction less than 20% are considered mechanically strong microneedles [45]. As illustrated in Figure 4e, the formulated LD/CD MAPs had a height reduction of 12.84 ± 2.34%, and the blank MAPs made from the same polymeric composition had a height reduction of 10.55 ± 3.88% (*n* = 5). The difference between the two investigated formulations is considered not significant (*p* > 0.05), presenting an acceptable needle strength against compression.

The insertion profiles of the formulated dissolving MAPs are illustrated in Figure 4f. Figure 4g–j provides representative microscopic images of each Parafilm M^®^ layer investigated in this study. The artificial Parafilm^®^ M insertion model provides a simple and low-cost alternative to porcine skin, with a similar insertion depth [28]. Both drug-loaded and drug-free control MAPs were able to penetrate through the third Parafilm^®^ M layer (42.58 ± 7.79% of the total needles for LD/CD MAPs, 57.29 ± 15.66% of the total needles for blank control MAPs), which was equivalent to approximately 378 µm, demonstrating a sufficient insertion depth to reach the dermal vasculature [46].

### 3.4. Dissolution Studies of the Formulated LD/CD Dissolving MAPs

**Figure 5:**
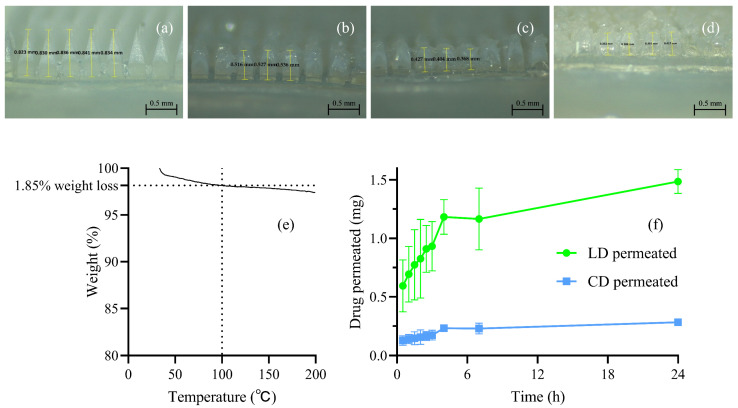
Digital microscopic images of LD/CD dissolving MAP dissolution upon insertion into PBS-soaked excised full-thickness neonatal porcine skin after (**a**) 0 min, (**b**) 15 min, (**c**) 30 min, and (**d**) 60 min. (**e**) TGA thermogram of the LD/CD dissolving MAPs. (**f**) Ex vivo permeation profiles of the LD/CD dissolving MAPs (mean ± S.D., *n* = 3).

The dissolution of the formulated dissolving MAPs was evaluated to understand the time required for the needles to be dissolved after application. Formulated MAPs were inserted into excised full-thickness neonatal porcine skin by manual force for 30 s and then transferred into a temperature-controlled oven which was set at 37 °C. Figure 5a–d displays the microscopic images of removed dissolving MAPs at 15 min, 30 min, 45 min, and 60 min. The needle length remaining reduced as the insertion time increased, and after 60 min, the vast majority of the white-cloudy ‘drug-containing’ part was dissolved and the ‘bump’ remaining on the baseplate was most likely the PVP 360 kDa ‘backing-support’ formulation, which takes a longer time to dissolve owing to the relatively high molecular weight. Therefore, the ‘drug-containing’ part of the dissolving MAPs was considered to be completely dissolved upon 1 h of application, leaving the incorporated cargo in the dermis for drug release.

### 3.5. Determination of the Drug Content of the Formulated LD/CD Dissolving MAPs

The drug contents of the formulated LD/CD dissolving MAPs were measured using the HPLC–UV method described in Section 2.6.1. The calculated drug contents of LD and CD per 0.5 cm^2^ area are 1.82 ± 0.24 mg and 0.47 ± 0.04 mg (*n* = 3). The ratio between LD and CD in the dissolving MAPs is approximately 3.87:1, which is in close agreement with the 4:1 ratio in the commercialised Rytary^®^ marketed tablet [4].

### 3.6. Determination of the Water Content of the LD/CD Dissolving MAP Final Product

A Thermal Advantage Model Q500 TGA TA Instruments, Elstree, Hertfordshire, UK) was used to quantify the water content left in the final MAP product. The weight loss at 100 °C was considered as the evaporated water content, which was less than 2% in this case, indicating that the 48 h drying process was thorough for water removal. The TGA graph is illustrated in Figure 5e.

### 3.7. Ex Vivo Skin Permeation Studies Using Dermatomed Neonatal Porcine Skin

Ex vivo LD/CD permeation across dermatomed neonatal porcine skin from dissolving MAPs was investigated to evaluate the transdermal drug delivery efficiency of the formulation. It has been previously proposed that dermatomed porcine skin is a suitable model for sole permeation investigation based on the distance from the SC to the dermis [46,47]. Figure 5f displays the permeation profile of an LD/CD dissolving MAP over a time period of 24 h. The drug permeation increased as a function of time, with a total permeation of 1.49 ± 0.10 mg/24 h for LD and 0.28 ± 0.02 mg/24 h for CD from the 1.82 ± 0.24 mg and 0.47 ± 0.04 mg LD and CD initial loading in one patch. A relatively high percentage of the drug (81.82% ± 5.66% LD, 60.15 ± 1.88% CD) was delivered to the receiver compartment after 24 h of MAP application. The relatively high percentage of drug delivery over a period of 24 h is due to the interstitial fluid uptake, hydration of polymeric chains, and subsequent dissolution to release the drug payload across the skin. The result conforms to previous published work (>60% drug loading) that utilised dissolving MAPs for transdermal delivery of hydrophilic compounds and it also reveals the successful implementation of the ‘two-layered’ dissolving MAPs to improve the drug delivery efficiency and avoid drug wastage, as previously mentioned [48].

While the ex vivo profile indicated a high amount of drug release over 24 h, it is unlikely to infer the pharmacokinetics in an in vivo work and predict the performance of the formulated dissolving MAPs in an in vivo condition, given that an acidic release media was selected to prevent LD degradation during the permeation study, which might alter the ionisation state of the skin and result in an overestimated permeation profile. Further in vivo pharmacokinetic study was carried out to investigate the drug release profile consequently.

### 3.8. In Vivo Pharmacokinetic Studies Using Healthy Sprague–Dawley^®^ Rats

The purpose of this in vivo study is to evaluate the pharmacokinetic profiles of the formulated MAPs and investigate any clinical benefit that can justify the necessity of using MAPs to deliver LD and CD simultaneously. The dose given to rats was calculated based on the ex vivo bioavailability calculated in Section 3.7. Four patches were applied to the backs of rats, and the oral suspension of LD/CD was prepared to have the same dose (which can be delivered) as the dissolving MAPs.

**Figure 6:**
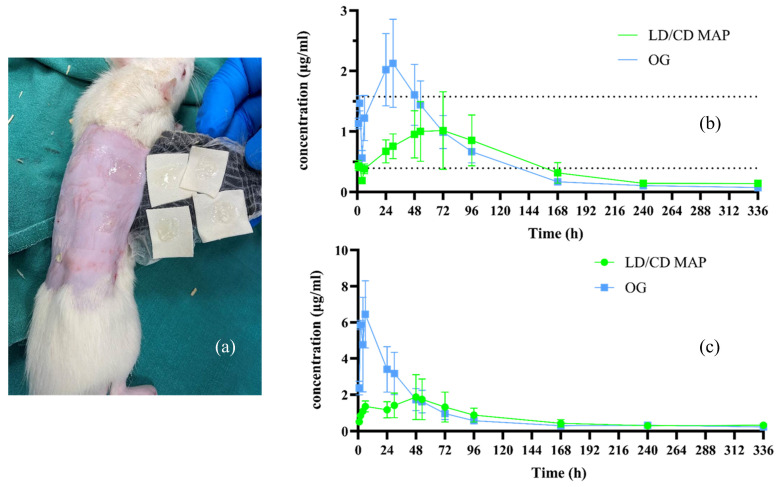
(**a**) Representative images of the backs of rats upon LD/CD MAP removal. In vivo pharmacokinetic profiles of (**b**) LD and (**c**) CD (means ± S.D., *n* = 3 for 1, 2, 4, and 6 h, *n* = 6 for the other sampling points). The dotted lines show the therapeutic window of LD [49].

As presented in Figure 6a, after 24 h of MAP application, the majority of the dissolving MAPs were dissolved, leaving only the high-molecular weight PVP K90 ‘supporting baseplate’ formulation on the removed Microfoam^TM^ tape. Blood samples were taken, processed, and analysed using the HPLC–fluorescence method described in Section 2.6.2. The in vivo pharmacokinetic profiles of LD and CD from both investigated formulations are illustrated in Figure 6b,c, respectively. As depicted in the graph, LD and CD were detected in the plasma 1 h after the application of the dissolving MAPs, demonstrating rapid dissolution and drug release. The plasma concentrations of CD gradually increased until 48 h (T_max_), at a mean concentration of 1.871 ± 1.232 µg/mL, indicating the formation of a polymer-based drug depot in the skin after the MAP application, whereas the CD plasma concentration of rats post-oral gavage administration started to decrease after 6 h. The orally administered LD showed a C_max_ of 2.129 ± 0.727 μg/mL at 30 h, whereas the C_max_ of LD (1.016 ± 0.639 μg/mL) from the dissolving MAPs occurred at 72 h. The delayed C_max_ of LD from the rats receiving the oral gavage could be attributed to a saturated metabolism, as a relatively high dose (22 mg/kg LD, 6 mg/kg CD) was matched to that of the rats receiving four MAPs. It has been reported that the minimum effective plasma concentration of LD is approximately 394.36 ng/mL (3 nmol/mL) [49]. The plasma levels of LD of rats treated with the dissolving MAPs stayed within the therapeutic range for at least 4 days, whereas those of the rats orally administered LD/CD were above the therapeutic range from 24 h to 48 h, highlighting the benefits of using the dissolving MAPs to avoid unnecessarily high plasma concentrations. The pharmacokinetic parameters were calculated and are presented in Table 3 and Table 4. The oral bioavailability of LD for rats has been reported to be approximately 62% [25]. Therefore, according to Equation (4), the relative bioavailability of LD from dissolving MAPs was calculated to be 37.22%, from which a difference in delivery efficiency was observed in comparison to the ex vivo permeation study performed in Section 3.7. One of the possible reasons was believed to be that the skin used in the Franz cell was more hydrated (12 mL release media), which led to an overestimation of the permeation across the skin ex vivo. Additionally, the acidic release media used potentially could also hasten the dissolution of both drugs and further resulted in a greater drug permeation than that in the in vivo study. Therefore, there could be the potential to keep the dissolving MAPs in situ for a longer period than 24 h to enhance the drug delivery.

There was no significant difference between the AUC values obtained from the oral control cohort (179.500 ± 14.390 µg·h/mL) and those of the LD/CD MAPs cohort (149.400 ± 21.970 µg·h/mL) (*p* > 0.05). However, a continuous release behavior was observed with the LD/CD dissolving MAPs, whereas the oral LD/CD formulation showed a rapid rise in plasma concentrations post-administration and reached C_max_ within 24 h. Furthermore, given that the gastric mobility of PD patients is commonly compromised, this pharmacokinetic study, carried out on healthy rats, may not be able to thoroughly reflect the potential benefits of using a non-oral formulation [9]. Therefore, the formulated LD/CD dissolving MAPs, in the other words, offer not only a great alternative approach that can bypass the gastrointestinal tract as well as first-pass metabolism in comparison to Sinemet^®^, but also a minimally invasive LD/CD formulation in comparison to Duodopa^®^ intestinal gel. Future work can focus on justifying the necessity of using dissolving MAPs to deliver LD and CD in the presence of gastric mobility issues. It would also be interesting to investigate the potential of achieving long-acting LD delivery using hydrogel-forming MAPs and keeping the patches on the skin for longer periods than 24 h [50].

## 4. Conclusions

This work introduced, for the first time, a dissolving MAP formulation that aimed to simultaneously deliver the PD gold standard medication, LD, and its decarboxylase inhibitor, CD. The formulated dissolving MAPs had sufficient mechanical strength and insertion abilities, showing promise in reaching the dermal vasculature upon application to the skin. A simultaneous delivery of both LD and CD from the dissolving MAPs was found in the ex vivo permeation studies using dermatomed neonatal porcine skin. The in vivo pharmacokinetic profile of the dissolving MAPs also presents continuous drug delivery and a comparable AUC value to that of the oral suspension through a transdermal administration route that can avoid the influence of gastric mobility issues in PD patients. Overall, the results indicate the promising role of the formulated dissolving LD/CD MAPs in delivering both compounds transdermally in a minimally invasive approach.

## Figures and Tables

**Table 1:** Experimental setup for the in vivo pharmacokinetic study.

Cohort	Dose
LD/CD MAP cohort	22 mg/kg LD, 6 mg/kg CD (estimated dose based on ex vivo studies)
LD/CD oral gavage	22 mg/kg LD, 6 mg/kg CD

**Table 2:** ‘Drug-containing’ layer formulation parameters of the LD/CD dissolving MAP.

Excipients	Percentage Composition (*w*/*w*)	pH
LD	20%	3.72
CD	5%	
PAA 250 kDa (35% *w*/*w* solution)	2.4%	
PVA 9–10 kDa (40% *w*/*w* solution)	39.6%	
Deionised water	33%	

**Table 3:** In vivo pharmacokinetic parameters of LD (*n* = 6).

	Parameter	LD/CD MAP	Oral Gavage
LD	C_max_ (μg/mL)	1.016 ± 0.639	2.129 ± 0.727
	T_max_ (hour)	72	30
	AUC (μg·h/mL)	149.400 ± 21.970	179.500 ± 14.390

**Table 4:** In vivo pharmacokinetic parameters of CD (*n* = 6).

	Parameter	LD/CD MAP	Oral Gavage
CD	C_max_ (μg/mL)	1.871 ± 1.232	6.443 ± 0.601
	T_max_ (hour)	48	6
	AUC (μg·h/mL)	232.900 ± 29.070	309.500 ± 28.250

## Data Availability

All data are included in the manuscript.
